# Pharmacological and non-pharmacological predictors of the LSD experience in healthy participants

**DOI:** 10.1038/s41398-024-03074-9

**Published:** 2024-09-04

**Authors:** Patrick Vizeli, Erich Studerus, Friederike Holze, Yasmin Schmid, Patrick C. Dolder, Laura Ley, Isabelle Straumann, Anna M. Becker, Felix Müller, Denis Arikci, Matthias E. Liechti

**Affiliations:** 1 Clinical Pharmacology and Toxicology, Department of Biomedicine and Department of Clinical Research, University Hospital Basel, Basel, Switzerland; 2 Department of Pharmaceutical Sciences, University of Basel, Basel, Switzerland; 3 University of Basel, Department of Psychology, University of Basel, Basel, Switzerland

**Keywords:** Clinical pharmacology, Predictive markers

## Abstract

The pharmacodynamic effects of lysergic acid diethylamide (LSD) are diverse and different in different individuals. Effects of other psychoactive substances have been shown to be critically influenced by non-pharmacological factors such as personality traits and mood states. The aim of this study was to determine pharmacological and psychological predictors of the LSD effects in healthy human subjects. This analysis is based on nine double-blind, placebo-controlled, cross-over studies with a total of 213 healthy subjects receiving between 25–200 µg LSD. The influence of sex, age, dose, body weight, pharmacogenetic, drug experience, personality, setting, and mood before drug intake on the peak autonomic and total subjective responses to LSD was investigated using multiple linear mixed effects models and Least Absolute Shrinkage and Selection Operator regression. Results were adjusted for LSD dose and corrected for multiple testing. LSD dose emerged as the most influential predictor, exhibiting a positive correlation with most response variables. Pre-drug mental states such as “Well-Being”, “Emotional Excitability”, and “Anxiety” were also important predictor for a range of subjective effects but also heart rate and body temperature. The trait “Openness to Experiences” was positively correlated with elevated ratings in “Oceanic Boundlessness” and mystical-type effects. Previous experiences with hallucinogens have been negatively associated with the overall altered state of consciousness and particularly with “Anxious Ego Dissolution”. Acute anxiety negatively correlated with the genetically determined functionality of the Cytochrome 2D6 enzyme. In summary, besides the amount of drug consumed, non-pharmacological factors such as personal traits and current mood also significantly predicted the subjective drug experience. Sex and body weight were not significant factors in influencing the drug experience.

## Introduction

Lysergic acid diethylamide (LSD) is a potent classic serotonergic psychedelic substance [1]. There has been renewed interest in the potential therapeutic applications of psychedelics such as psilocybin and LSD, especially for depressive, anxiety, and substance use disorders [2–8].

The acute effects of LSD can encompass profound shifts in consciousness, audio-visual synaesthesia, mystical/spiritual experiences, and increased introspection [9–12]. The response to LSD is dose-dependent [11], similar to other classic serotonergic psychedelics like psilocybin or mescaline [10, 13, 14]. However, consistent with the inherent variability observed in response to psychoactive drugs, inter- and intra-individual differences in the response can manifest even under equivalent dosing regimens, indicating a role for non-pharmacological factors, typically framed as “set” and “setting” [15–18]. Past studies have explored “set” and “setting” aspects in studies with psilocybin and the entactogen 3,4-methylenedioxymethamphetamine (MDMA) [19–21]. However, modern studies on LSD effect modulators are lacking. Additionally, it has previously been shown that individual differences in the genes coding for enzymes involved in the metabolism of LSD (i.e., Cytochrome P450 2D6 (CYP2D6)) influence exposure to LSD and can thereby also affect its acute effects [22, 23].

Previously recorded pharmacological and non-pharmacological factors can serve as potential indicators of the expected response to LSD. This has already been preliminarily documented for psychedelic drugs [24–26], and in greater detail for psilocybin [21] and MDMA [20]. It is plausible that these substances share, at least to some extent, analogous modulatory determinants for their effects.

As psychedelics, including LSD, are increasingly discussed as potential new therapeutic substances for the treatment of various mental disorders, it is crucial to understand their effects in more detail. Intriguingly, the quality of the acute psychedelic experience has been observed to predict the therapeutic outcome, and self-reported positively experienced effects and mystical-type experiences have been associated with better long-term treatment response [3, 27–29]. Thus, it is crucial to understand the factors that optimize the quality of the acute psychedelic effects to enhance patient selection, preparation processes, and potentially therapeutic outcomes.

This study therefore aimed to examine the influence of several predictor variables on acute physiological and psychological responses in healthy human subjects to doses of LSD ranging from 25 to 200 µg. To the best of our knowledge, this represents the largest dataset featuring a uniformly collected array of predictor and outcome variables for psychedelic effects. Additionally, this is the first study to evaluate predictors of the LSD experience that accounts for the actual administered dosage and displays the importance of different variables.

## Methods and materials

### Study design

This is a pooled analysis of the raw data from nine double-blind, mostly placebo-controlled (8/9), crossover studies in healthy human participants, eight of which have been previously described [9–11, 13, 23, 30–32]. The studies were all registered at ClinicalTrials.gov (Study #1: NCT01878942, #2: NCT02308969, #3: NCT03019822, #4: NCT03321136, #5: NCT03604744, #6: NCT04227756, #7: NCT04516902, #8: NCT04558294, #9: NCT04865653). Written informed consent was obtained from all participants. The studies were conducted at the University Hospital Basel from 2014 to 2023 and included a total of 213 healthy subjects, in 297 LSD-only sessions and 189 placebo sessions. Some studies included conditions with LSD plus another substance. In the present analysis, only data from the LSD-alone (*N* = 297) and placebo (*N* = 189) sessions were used. Study #8 [30] did not include a placebo session. Details are shown in the Supplemental Material.

### Subjects

A total of 213 (108 female) healthy subjects, aged 25–64 years (mean ± SD = 32 ± 9 years), were recruited from the campus of the University of Basel and participated in the study. The mean ± SD body weight was 70 ± 12 kg (range: 50–104 kg). A detailed summary of the included study population is provided in Supplementary Table S1. Exclusion criteria are in the Supplemental Material.

### Study drug

LSD base (D-lysergic acid diethylamide, Lipomed AG, Arlesheim, Switzerland) was administered orally at a single dose of 25, 50, 100, and 200 µg prepared as gelatine capsules [9, 32] or as drinking solution ([10, 11, 13, 23, 30, 31], NCT04865653) in flasks that contained 25 or 100 µg LSD in 1 mL of 96% ethanol. Content uniformity and the analytically confirmed amount of LSD freebase were available for all but the first two studies [33]. The actual doses of the first two studies were estimated based on the area-under-the-curve (AUC) of the LSD blood plasma concentration levels as described elsewhere [22]. Only the LSD base condition of Study #9 was included in the present analysis. Analytically confirmed doses were between 26 and 197 µg (Supplementary Table S1).

### Predictor variables

Predictor variables included: sex, age, drug dose (also as covariate, see statistical analysis), body weight, genetical predisposition of CYP2D6 enzyme, the number of previous hallucinogenic experiences, undergoing of a magnetic resonance imaging (MRI) procedure, subjective mood prior to drug intake measured by the Adjective Mood Rating Scale (AMRS) [34], and the NEO Five-Factor Inventory (NEO-FFI) [35]. Details in the Supplemental Material. Histograms of the predictor variables are presented in Supplementary Fig. S1A.

### Response variables

Response variables included Visual Analog Scales (VASs), the five dimensional Altered States of Consciousness (5D-ASC) [36, 37], the 30-item Mystical Experience Questionnaire (MEQ30) [38], mean arterial blood pressure (MAP), heart rate, body temperature, and the area under the LSD blood plasma concentration curve from time zero to infinity. For subjective responses, we utilized the area under the effect-time curve (AUEC) to quantify the overall acute drug effect throughout the study day (0–11.5 h). Physiological variables were analyzed using the peak change (*E*_max_) from placebo, reflecting the maximum observed drug induced change during the assessment period. All response variables are difference values between LSD and the respective placebo session. More details in the Supplemental Material. Histograms of the response variables are presented in Supplementary Fig. S1B.

### Statistical analyses

Response variables were analyzed as the difference of LSD from the placebo session. All data were analyzed using the R language and environment for statistical computing [39]. Some of the predictor and response variables contained missing data which were imputed differently (shown in Supplemental Material and Table S2).

To account for the clustering in our data arising from multiple data points from the same subject because of multiple doses tested within study #4 and #5, we used linear mixed effects models in which the intercepts were allowed to vary randomly across subjects. Additionally, we conducted a supportive sensitivity analysis in a dataset with only one dose per study (i.e., the dose closest to 100 µg) to support our main analysis (shown in Supplementary Fig. S2). For each combination of predictor and response variable, an adjusted model was fitted using the R package nlme [40]. Since LSD displays clear dose-dependent effects [11], the actual dose administered was additionally included in the fixed effects part (except when analyzing the influence of the drug dose). Before being included in the models, both predictor and response variables were z-transformed. This ensured that the estimated regression coefficients were fully standardized, allowing comparability between the different predictors and responses.

To account for multiple testing, *p*-values were corrected (p_c_) across all significance tests using the Benjamini-Hochberg procedure [41]. In each linear mixed effects model, the amount of variance explained by each fixed effects predictor was determined by calculating the semi-partial *R*^2^ (*sr*^2^) using r2beta function in the r2glmm package.

To determine the optimal subset of predictors for each response variable and to assess the relative importance of these predictors, we employed the least absolute shrinkage and selection operator (LASSO) technique using the R package “penalized” [42] (see Supplemental Material for details). Additionally, to explore the shared explained variance of all variables and certain subsets, such as readily available demographic data, further full model approaches were investigated, which are shown in Supplementary Fig. S3.

## Results

**Fig. 1:**
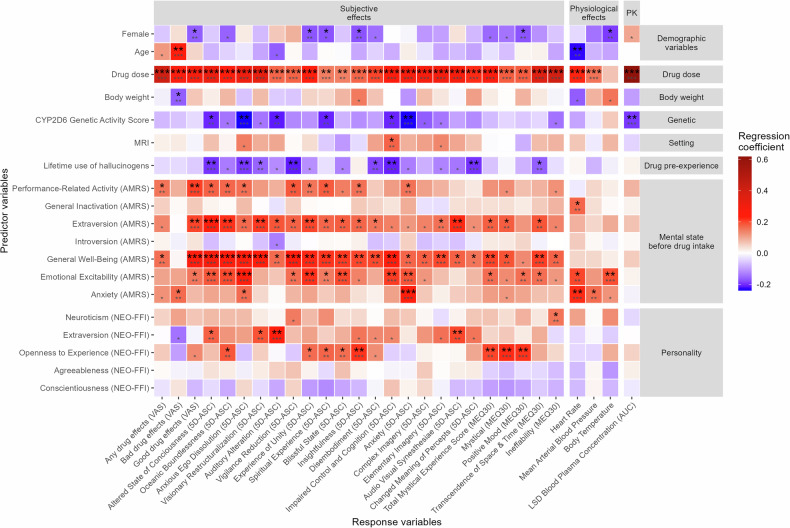
Standardized regression coefficients and statistical significance of each predictor variable in the linear mixed effects models adjusting for drug dose (except drug dose). The data used are the difference between the LSD and the respective placebo session. Smaller asterisks show the uncorrected statistical significance. Bigger asterisks show the significance after correction for multiple testing across all 19 * 29 = 551 significance tests using the Benjamini-Hochberg procedure [41]. *p < 0.05, **p < 0.01, ***p < 0.001. *N* = 297. The *p*eak effect was used for the physiological effects. CYP cytochrome P450, MRI magnetic resonance imaging, VAS visual analog scale (area under the effect-time curve 0–11.5 h), AMRS adjective mood rating scale, NEO-FFI NEO five-factor inventory, 5D-ASC five dimensional altered states of consciousness, MEQ30 30-item mystical effects questionnaire, AUC area under the curve from 0–∞h. Detailed statistical estimates are listed in Supplementary Table S4.

The magnitude of the fully standardized regression coefficients, *sr*^2^, and the statistical significance of each predictor variable for each outcome variable are shown in Fig. 1 and reported in Supplementary Table S4.

The amount of LSD administered (26–197 µg, Supplementary Table S1) was the strongest predictor of all LSD-induced effects except for body temperature. Body temperature was only predicted by sex (female participants had smaller temperature changes from placebo) and pre-drug “Emotional excitability“ as measured by the AMRS. Among the most statistically significant associations observed with LSD dose were LSD blood plasma concentration, followed by VAS “any drug effect”, and 5D-ASC total score. The only other predictor of LSD blood plasma concentration was the CYP2D6 activity score. The CYP2D6 activity score was also negatively associated with the 5D-ASC total score and, in particular, with “Anxious Ego Dissolution,which includes “Anxiety” and “Impaired Control and Cognition”. In LSD sessions involving MRI measurements participants reported more “Impaired Control and Cognition” on the 5D-ASC. The participant’s age was positively, and body weight was negatively associated with VAS “bad drug” effect.

Female sex was associated with smaller LSD-induced increases in VAS “good drug effect”, 5D-ASC “Experience of Unity”, “Insightfulness”, and MEQ30 “Positive Mood”. Prior experience with hallucinogens predicted less overall altered state of consciousness (5D-ASC total score) and particularly effects like “Anxious Ego Dissolution”, “Vigilance Reduction”, “Disembodiment”, “Impaired Control and Cognition”, “Changed Meaning of Percepts”, and MEQ30’s “Transcendence of Space and Time”.

Apart from drug dose, mental mood state before drug intake, specifically “General Well-Being” was the second strongest predictor of subjective effects of LSD. It showed significant associations with all subjective effects, except for VAS “bad drug effect”, 5D-ASC “Changed Meaning of Percepts”, and MEQ30 “Positive Mood”. Among the most statistically significant association observed with “General Well-Being” was the 5D-ASC total score, followed by the VAS scale “good drug effect”, and “Oceanic Boundlessness” in the 5D-ASC. “Performance-Related Activity”, “Emotional Excitability”, “Extraversion”, and “Anxiety” before drug intake were other important predictors for several subjective effects (shown in Fig. 1). “Emotional Excitability”, “General Inactivation”, and “Anxiety” also significantly predicted heart rate. AMRS “Anxiety” before drug intake also predicted higher scores in the 5D-ASC subscale “Anxiety” during the experience. State “Introversion” did not predict any response variable.

The personality trait “Openness to Experience” captured by the NEO-FFI questionnaire predicted 5D-ASC “Oceanic Boundlessness”, “Spiritual Experience”, and “Insightfulness” and the total MEQ30 score, as well as the MEQ30 subscales “Mystical”, and “Positive Mood”. Furthermore, the trait “Extraversion” was positively associated with the 5D-ASC total score and the subscales “Visionary Restructuralization”, “Auditory Alteration”, and “Audio Visual Synesthesia”. The character traits “Neuroticism”, “Agreeableness”, and “Conscientiousness” did not predict any LSD effects.

The results were additionally tested in a smaller subset including only one-dose per study (doses close to 100 µg LSD, *N* = 213, shown in Supplementary Results and Fig. S2) as a sensitivity analysis.

**Fig. 2:**
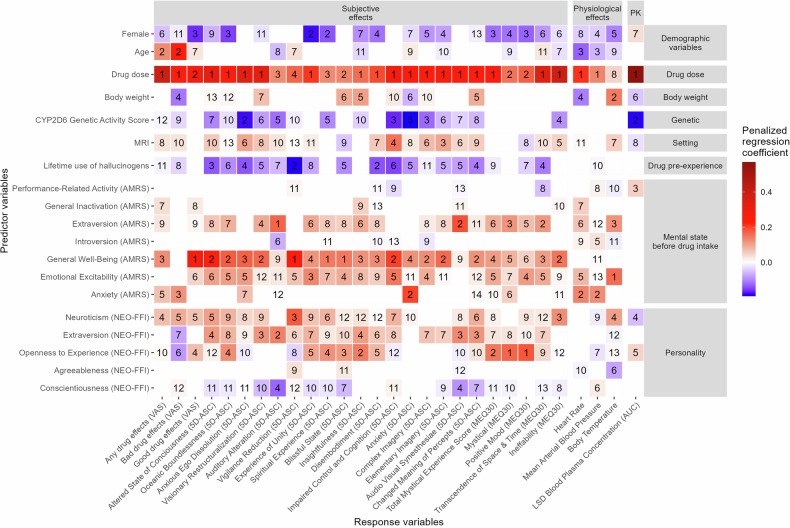
Size of the penalized regression coefficients and rank of importance of the predictor variables in the least absolute shrinkage and selection operator (LASSO) models. As one LASSO model was developed for each response variable, each column in the tile plot displays the results of one LASSO model. The rank of relative importance of each predictor for each outcome was determined by ranking the predictor variables according to their absolute size of the regression coefficients in each LASSO model. The data used are the difference between the LSD and the respective placebo session. The peak effect was used for the physiological effects. CYP cytochrome P450, MRI magnetic resonance imaging, VAS visual analog scale (area under the effect-time curve 0–11.5 h), AMRS adjective mood rating scale, NEO-FFI NEO five-factor inventory, 5D-ASC five dimensional altered states of consciousness, MEQ30 30-item mystical effects questionnaire, AUC area under the curve from 0–∞ h.

The penalized regression coefficients of the LASSO models are shown and ranked in Fig. 2. On average, 12 predictors (range: 8–14) were selected for each response variable. The drug dose administered was selected as predictor for all the response variables. It had the largest absolute standardized regression coefficient in 21 of 29 response variables and thus was the most important predictor. However, while it was the most important predictor for most LSD effects, it was not the most important one for “good drug” effects, “Auditory Alteration”, “Vigilance Reduction”, “Spiritual Experience”, “Blissful State”, “Mystical”, “Positive Mood”, or body temperature. Specifically, the most important predictor for the VAS “good drug” effects, 5D-ASC “Vigilance Reduction”, “Spiritual Experience”, and “Blissful State” was “Well-Being” before drug intake while it was “Extraversion“ in the NEO-FFI for “Auditory Alteration”, and “Emotional Excitability” before drug intake for body temperature. Furthermore, the character trait “Openness to Experience” was the most important predictor for “Mystical” and “Positive Mood” effects in the MEQ30 and the second most important predictor for 5D-ASC “Insightfulness”. “Anxiety” before drug intake was the second most important predictor for 5D-ASC “Anxiety”, heart rate, and MAP.

## Discussion

The present study examined the influence of 19 predictor variables on subjective psychological and autonomic responses to LSD in healthy human subjects. The amount of drug administered was the most important predictor for most acute LSD effects. However, when adjusted for the drug dose, other predictor variables such as pre-drug mood, CYP2D6 genotype, age, prior experience with hallucinogens and personality traits had a distinct influence on the physiological and psychological response to LSD. The genetic activity of CYP2D6 was the only other predictor besides drug dose that was associated with LSD blood plasma concentration. Lower CYP2D6 activity scores were also associated with a higher overall 5D-ASC score and particularly more “Anxious Ego Dissolution,” which includes “Anxiety” and “Impaired Control and Cognition”. Previous experience with hallucinogens has been associated with lower overall 5D-ASC scores, and in particular with reduced “Anxious Ego Dissolution” and “Impaired Control and Cognition”. Consistent with psilocybin studies [21], where hallucinogen-naïve individuals experienced more “Disembodiment”, “Visionary Restructuralizaion”, and “Changed Meaning of Percepts” in our study prior experience with hallucinogens predicted less effects in these 5D-ASC items. Furthermore, vital signs were equally influenced by drug dose, but also by pre-drug emotional excitability and anxiety. The subjective effects were more pronounced if the subject showed higher ratings of “Well-Being”, “Extraversion”, “Emotional Excitability”, “Activity”, or “Anxiety” before drug intake. Additionally, participants with higher scores in personality trait “Openness to Experience” had a more insightful and mystical experience, both on the 5D-ASC and the MEQ30 questionnaire. Meanwhile, a higher score on the “Extraversion” personality trait predicted more visual and auditory effects. Female participants generally reported a slightly less positive experience than their male counterparts; however, this difference was not statistically significant in the sensitivity analysis (shown in Supplementary Fig. S2). Meanwhile, older subjects tended to experience more “Bad Drug Effects” during the study day. Factors such as body weight and setting (MRI) had a smaller impact on the evaluated response variables.

The analysis revealed that the dose of LSD administered is the primary predictor of its effects and stands as the most significant overall determinant. This dose-dependent response is in line with previous study findings [11, 43–45]. However, a recent meta-analysis, also highlighted the significant role of non-pharmacological factors in shaping subjective experiences [45]. Not only are the subjective effects influenced by the non-pharmacological factors, but the autonomic response also appears to depend equally on both, the drug dose and emotional state prior to intake. The moderate impact of dosage on autonomic responses implies that LSD’s primary influence on these effects stems from psychological arousal rather than from direct cardiostimulant properties of LSD consistent with its cardiovascular safety [46]. Adverse effects of psychedelics are often equated with challenging experiences, commonly referred to as “bad trips,” rather than physical complications [17, 47]. In our study, older age emerged as the second most significant predictor of “bad drug effects”. This finding is surprising, especially when contrasted with other studies on psilocybin, in which younger age was correlated with more challenging experiences [21, 48]. Nonetheless, it is important to note that the “Bad Drug Effects”, while present, were generally small and clearly relatively smaller than the “Good Drug Effects”. Moreover, these “bad” effects encompassed not only psychologically challenging experiences but any negative state, including nausea. In fact, in our study, challenging experiences - such as “Anxious Ego Dissolution” or “Anxiety” as captured in the 5D-ASC and highlighted in studies by Studerus et al. and Ko et al. [21, 48]—were not significantly associated with age. Another previously determined predictor of challenging experiences was the personality trait “Neuroticism” [49]. However, in our study, “Neuroticism” did not show a pronounced influence on the effects of LSD. In a previous study, it has been reported that subjects experience more challenging experiences if they must undergo positron emission tomography (PET) during the effects of psilocybin [21]. In our study with LSD, we examined the effects of MRI scans as opposed to PET. In contrast to PET, we found that those undergoing an MRI scan reported an increase in “Elementary Imagery”, but did not exhibit heightened anxiety. We identified “Anxiety”, including feelings of anticipation, anxiety, and being nervous prior to substance administration significantly predicted challenging effects. This is consistent with a previous prospective web-based survey, in which feeling adequately prepared and ready before drug intake was predictive of less challenging experience in response to various psychedelics [24]. In contrast, this association was not observed in studies with psilocybin [21]. However, both “Emotional Excitability” and “Activity” were predictors of higher scores in the 5D-ASC for both LSD and psilocybin. Notably, in our sample, “Well-Being”, which includes subscales such as feelings of happiness, satisfaction and self-confidence prior to drug administration emerged as the paramount non-pharmacological predictor for subjective effects. This was also not observed with psilocybin, as shown in a prior study by Studerus [21].

The present study confirmed that personality traits, particularly “Extraversion” and “Openness to Experience”, have a significant influence on the subjective response to LSD. Participants who ranked higher on “Extraversion” consistently reported increased scores on the 5D-ASC, with an emphasis on visual and auditory alterations. This finding was previously also reported for psilocybin [21]. “Sociability” in the Zuckerman-Kuhlman Personality Questionnaire, which is highly convergent with “Extraversion” [50], likewise predicted more “Audio Visual Synesthesiae” after psilocybin [21]. One possible explanation is that those scoring lower in “Extraversion”, being typically more oriented to internal stimuli, could be less focused on external cues, making them less perceptive to nuanced changes. “Openness to Experience” was particularly associated with more positive experiences, such as “Oceanic Boundlessness”, “Insightfulness”, and mystical-type experiences. As described in the 5D-ASC questionnaire, “Oceanic Boundlessness” characterises the uplifting dimensions of the experience, encompassing a sense of “oneness with oneself and the universe”, as well as a freedom from spatial and temporal constraints. This finding is consistent with many previous observations where the personality trait of “Absorption”, which conceptually overlaps strongly with the personality trait of “Openness to Experience” [51], has been identified as a key predictor of both pleasurable and mystical experiences following the use of psychedelics [24, 52] including psilocybin [21, 53], ayahuasca [54], and the entactogen MDMA [20].

Importantly, many different studies indicated that elevated 5D-ASC “Oceanic Boundlessness” scores and psychedelic-induced mystical-type experiences captured by the MEQ30 are correlated with favourable long-term clinical outcomes [3, 5, 27, 29, 55, 56], whereas negative effects, such as “Anxious Ego Dissolution”, had no effect [3] or were correlated negatively [27] with the change in depressive symptoms following psychedelic-assisted psychotherapy. Moreover, a recent review on the therapeutic use of psychedelic substances for various mental disorders highlighted that the intensity of the acute psychedelic experiences is a paramount predictor of therapeutic response [57].

Considering the aforementioned findings, it can be inferred that the acute effects are profoundly influenced by the individual’s pre-drug mental disposition and inherent personality traits and might therefore alter the clinical efficacy of psychedelics. These factors are frequently embodied in the conceptual framework referred to as “set”. This is particularly noteworthy when juxtaposed with the entactogen MDMA, wherein “set” was a far less influential predictor [20]. This observation suggests that enhancing an individual’s mood, perhaps through the use of less “set”-dependent substances and robust inducers of positive mood like MDMA, prior to administering a psychedelic could prove beneficial, even though simultaneous application did not [23].

“Well-Being” and positive persisting effects were also increased two to four weeks [24], three month [58], or even up to a year [59] after a psychedelic experience and this was consistently predicted by mystical-type experiences [24, 58, 59]. Moreover, openness and partly extraversion has been shown to increase in a similar way after a psychedelic experience, as demonstrated in studies using psilocybin or LSD [60–62] or MDMA [63]. One might therefore speculate that another way to enhance these predictors, i.e., openness and “Well-Being”, prior to a psychedelic treatment could be by conducting multiple sessions. Indeed, this has been also the conclusion of a recent review [64] and has already been done in several phase II trials with psychedelics [3, 7] and phase III trials with MDMA [65, 66]. As mentioned earlier, MDMA could serve not only as a preparatory enhancement but also as an introductory psychedelic-like experience. However, it is unlikely to prove superior to a modest introductory dose of a classic psychedelic. The significant impact of dosage cannot be overlooked; hence, it is important to consider that a dose escalation should ideally occur when a patient is more open to the effects. This hypothesis is further supported by our findings indicating that prior experience with hallucinogens predicts lower scores on negatively associated effects, such as “Anxious Ego Dissolution”. This supports the use of ascending dosing regimens which have already been implemented and tested in some studies with classical psychedelic but also MDMA ([65–69], and upcoming trials with LSD: NCT03866252, NCT05883540).

While the present study provides a unique and comprehensive overview of the potential factors influencing psychological and physiological responses to LSD in healthy individuals in a controlled environment, it also has some limitations. First, although all but one of the analyzed studies included a placebo session, blinding is a recognized problem with psychoactive substances [70] and expectations potentially influence subjective effects.

Secondly, while highly standardized laboratory study designs ensure the high quality of data collected, the “physical and social environment,” often referred to as “setting” and representing a notable modifier of the response to psychedelics [16, 18], exhibited limited variation. In this study, participants were mostly situated in a bed within a quiet hospital room with a single investigator present. Hence, the only predictor in the domain of setting that could be used in the present study was MRI (i.e. whether subjects underwent an MRI during the effect of the drug). For recreational or therapeutic use, the “setting” may be quite different [71]. Another “setting” factor that was mostly present during the study sessions was music. We did not restrict or assess what kind or how much music the participants listened to; however, it has been demonstrated that at least, the genre of music might be irrelevant [72]. Thirdly, individuals with lower CYP2D6 activity scores had higher scores on 5D-ASC scales including “Anxious Ego Dissolution” which is consistent with higher LSD concentrations in persons with impaired CYP2D6 function. In addition, while the effect of CYP2D6 gene variations are likely mediated by the PK, variations in the serotonin 2 A receptor gene which is coding for the target receptor of LSD may also alter the effects of LSD, but were not investigated in the present study. Fourthly, psychedelic studies may attract individuals scoring higher on the personality trait “Openness to Experience” compared to the general or patient population. Fifth, while LASSO is effective for variable selection and regularization, it may not provide a clear rank-order of importance when predictors explain comparable degrees of variance. Additionally, the assumed linear dose-response relationship in the model might not hold true for all responses due to potential plateau effects [11]. However, our sensitivity analysis largely indicated linear dose-response relationships across the outcomes, and the results did not significantly deviate from our main analysis. Additionally, analyses using restricted cubic spline dose-response relationships were tested and yielded largely the same results. Finally, although an extraverted disposition, open-mindedness, and a positive mood prior to drug administration may potentially serve as predictors for enhanced acute and long-term effects as observed in healthy subjects, this might be difficult to achieve in patients with severe mental disorders. Further research is needed to explore how therapeutic interventions can optimize outcomes in clinical populations with potentially lower baseline well-being, higher anxiety, or in more elderly individuals. Therapeutic support, preparatory sessions or pharmacotherapy, could be employed to improve well-being and to reduce anxiety prior to psychedelic therapy. However, challenging experiences triggered by the drug may also harbour transformative potential, but may require more time or additional therapeutic interventions to fully unfold and be interpreted [73].

In summary, the present study underscores that, in addition to the amount of drug consumed, non-pharmacological factors such as subjective mood prior to drug use and the personality trait “Openness to Experience” play a central role in shaping the acute response to LSD. Conversely, sex and body weight exhibited no significant influence on the drug experience. With growing interest and use of LSD in research and psychotherapy, it is important to identify predictors to better prepare for the expected psychedelic response. Psychedelic experiences have the potential to be life-changing and very challenging [74], so having well-prepared individuals is crucial for positive clinical outcomes. By understanding and reinforcing these predictors towards the potential positive effects like “Oceanic Boundlessness” and mystical-type experiences, we may not only bolster therapeutic efficacy but also reduce the likelihood of adverse effects.

## Supplementary information

Supplemental Material

## Data Availability

The datasets presented in this article are not readily available because the data associated with this work are owned by the University Hospital Basel and were licensed by Mind Medicine. Requests to access the datasets should be directed to ML, matthias.liechti@usb.ch.
