# Supplementary material for: Pharmacological and non-pharmacological predictors of the LSD experience in healthy participants
Source: Transl Psychiatry. 2024 Sep 4;14:357. doi: 10.1038/s41398-024-03074-9 (PMC11374807; doi:10.1038/s41398-024-03074-9)
Supplement: Supplementary file 1 — Supplemental Material [file 41398_2024_3074_MOESM1_ESM.docx]

***Supplemental Material***

**Pharmacological and Non-Pharmacological Predictors of the LSD Experience in Healthy Participants**

This appendix includes supplemental methods, and results including tables and a figure.

# Methods and Materials

## **Study design**

In all of the pooled studies, the washout periods between the study sessions were at least 10 days to exclude carry-over effects. Test sessions were conducted in a quiet hospital research ward with no more than one research subject present per session. The participants were comfortably lying in hospital beds and were mostly listening to music and not engaging in physical activities. LSD was administered after a standardized breakfast in the morning at 8:00−10:00 AM. All the studies were approved by the local ethics committee and conducted in accordance with the Declaration of Helsinki. The use of LSD was authorized by the Swiss Federal Office for Public Health (BAG), Bern, Switzerland. Written informed consent was obtained from all participants. All participants were paid for their participation.

## **Subjects**

Exclusion criteria included a history of psychiatric disorders, physical illness, lifetime history of illicit drug use more than twenty times (except for past cannabis use, in Study #8 and #9, the restriction of lifetime use of illicit drugs was limited to hallucinogens), illicit drug use within the past 2 months, and illicit drug use during the study. Drug screens were conducted before the test sessions.

## **Predictor** **variables**

Sex was included because sex differences were reported for psychoactive substances like MDMA [1, 2]. Older age has been associated with more pleasant acute effects of psilocybin [3], while no data is available on LSD.

In vitro studies have shown that cytochrome P450 (CYP) enzymes (i.e., CYP2D6, CYP3A4, etc.) are involved in the metabolism of LSD [4, 5]. The activity of these enzymes can be significantly influenced by various genetic predispositions. In particular, CYP2D6 is associated with phenotypes ranging from fully functional to non-functional [6]. Individual differences in the genes for enzymes involved in the metabolism of LSD influence exposure to LSD and may also affect its acute effects [7]. Thus, the genetically determined activity score of the CYP2D6 functionality was included as predictor variable [8]. However, activity scores should be interpreted with caution. While they provide a quantifiable approach to predicting drug metabolizing ability based on genotype, linearity of the actual phenotype can be artificial and may not be given [9]. To clarify the data further, we provide an overview of the LSD plasma concentration and CYP2D6 activity score, as well as a comparison between functional and non-functional genotypes, as noted in a previous publication [7] (Supplementary Figure S4). Genotyping samples from 27 participants were not available because they did not consent to genotyping or the genotyping sample was not collected or analyzed (i.e. Study #9).

The number of previous hallucinogenic experiences was included as predictor variable in the present analysis, although subjects in the present studies had only limited previous experience with hallucinogenic substances (0-10 times, see Supplementary Table S1). Participants who had no previous experience with hallucinogenic substances showed higher ratings on some subscales of the five dimensional Altered States of Consciousness (5D-ASC) questionnaire after administration of psilocybin [3]. For the analysis we additionally included previous experience with hallucinogens during sessions within the respective cross-over study. In detail, this included NCT03321136, NCT03604744, NCT04227756, NCT04516902, and NCT04865653. Sessions, with only 25 µq LSD or when the effect was blocked (i.e. combination with ketanserin) were not counted as hallucinogenic experience.

Because the studies were all conducted in a very similar manner, only the magnetic resonance imaging (MRI) measurement at the time of peak experience could be used as a "setting" predictor variable.

The subjective mood state prior to the administration of a psychoactive substance influences its subjective effects, as has been shown previously for psilocybin and MDMA in similar analyses [3, 10]. Therefore, we similarly included ratings on the Adjective Mood Rating Scale (AMRS) [11] obtained just prior to LSD administration. The AMRS consists of 60 adjectives that are rated on a 4-point Likert scale and items were grouped into the subscales: “Performance-Related Activity”, “General Inactivation”, “Extraversion”, “Introversion”, “General Well-Being”, “Emotional Excitability”, and “Anxiety”. For this analysis, the “General Well-Being” score, which includes subscales such as feeling happy, satisfied, and self-confident was particularly relevant. Items included: “funny”, “confident”, “complacent”, “cheerful”, “carefree”, “joyful”, “glad”, and “self-confident”.

Personality traits were assessed as part of the screening procedure at baseline. The NEO-FFI [12] was applied, which contains 60 self-referent statements rated on a 5-point Likert scale. The NEO-FFI surveys the personality traits “Neuroticism”, “Extraversion”, “Openness to Experience”, “Agreeableness”, and “Conscientiousness”.

## **Response** **variables**

All response variables are difference values between LSD and the respective placebo session.The subjective response to LSD was assessed using psychometric scales. Visual Analog Scales (VASs) were used repeatedly from before and 0 to 11.5 - 24 hours after administration of LSD or placebo. VASs for “any drug effect”, “good drug effect”, and “bad drug effect” were presented as 100-mm horizontal lines (0–100%), marked from “not at all” on the left to “extremely” on the right. The responses on each VASs were included into the analysis as trapezoidal-calculated area under the effect-time-curve (AUEC) value, reflecting the overall response throughout the study day. Only the acute effects from 0 to 11.5 hours were included in the calculation to be consistent across studies (see Supplementary Table S1).

The subjective five dimensional Altered States of Consciousness (5D-ASC) questionnaire [13] was administered at the end of each study session to retrospectively assess alterations in waking consciousness. The 5D-ASC consists of 94 visual analog scale items and measures three etiology-independent and two etiology-dependent dimensions of altered states of consciousness. This questionnaire is one of the best validated instruments to capture subjective effects of psychoactive substances [3]. Furthermore, since these main dimensions are heterogenous constructs [14], we also included the more homogeneous subscales constructed by Studerus et al. [14].

The 30-item Mystical Experience Questionnaire (MEQ30) [15] was also assessed at the end of each study session. The questionnaire encloses 30 items that are included in the States of Consciousness Questionnaire (SOCQ) [16, 17]. The MEQ30 asks about mystical effects and is divided into four factors: mystical, positive mood, transcendence of time and space, and ineffability. While the 5D-ASC scale covers many of the different facets of altered states of consciousness and is most widely used in psychedelic research, the MEQ30 is better at capturing some mystical-type aspects and has often been used to predict positive long-term outcomes [18-21].

Blood pressure, heart rate, and body temperature were repeatedly assessed from before and from 0 to 11.5 - 24 hours after administration of LSD or placebo. Systolic and diastolic blood pressure and heart rate were measured using an automatic oscillometric device (OMRON Healthcare Europe NA, Hoofddorp, The Netherlands). Core (tympanic) temperature was measured using a GeniusTM 2 ear thermometer (Tyco Healthcare Group LP, Watertown, NY, USA). The mean arterial pressure (MAP) was calculated as diastolic blood pressure + (systolic blood pressure – diastolic blood pressure)/3. For our analysis, we selected the highest values (Emax) as the outcome variable for physiological response measures. This selection was made as elevated cardiovascular peak stimulation and body temperature are the clinically relevant, potentially adverse outcomes of interest.

Blood samples for plasma concentration measurements were collected in lithium heparin tubes repeatedly from before and 0 to 11.5 - 24 hours after administration of LSD or placebo. Blood samples were immediately centrifuged and the plasma was stored below -20°C until analysis. Plasma concentrations of LSD were determined as previously described [22]. The area under the concentration-time curve (AUC) from time zero to infinity was derived from non-compartmental analysis performed using Phoenix WinNonlin 8.3 (Certara, Princeton, NJ, USA).

## **Statistical analyses**

Study #8 did not include a placebo session and was subsequently not used for the analysis involving vital parameters. Study #2 did not assess the MEQ30 and was therefore not used in the analysis for predicting the MEQ30. Missing genotyping data was also not imputed. Subjective effects in missing placebo sessions were replaced with “0”. For all other missing values, we performed multiple imputation using the Multivariate Imputation via Chained Equations (“MICE”) package in R [23]. We opted for this method as it generates unbiased parameter estimates and standard errors under both "missing at random" (MAR) and "missing completely at random" (MCAR) missing data mechanism. Moreover, it maximizes statistical power by utilizing all available information [24]. The MAR assumption was plausible in this study because the missing data mostly resulted from random drop-outs or missing questionnaires. We generated 20 imputations for the missing values, resulting in 20 complete datasets. This approach was taken to prevent any potential decline in power due to an insufficient number of imputations [25]. Except for the LASSO models (see below), the analyses of interest were subsequently conducted in each completed dataset, and the parameter estimates were pooled in accordance with Rubin's rules [26].

In each linear mixed effects models, the amount of variance explained by each fixed effects predictor was determined by calculating the semi-partial *R^2^* (*sr^2^*) using r2beta function in the r2glmm package. The reported *sr^2^* values therefore only describe the unique contribution of a single independent variable to the variance explained in the dependent variable, after accounting for the variance explained by dose, but not by any other predictor variables.

Least absolute shrinkage and selection operator (LASSO) performs both variable selection and regularization, which involves shrinking the regression coefficients and aims to improve the predictive accuracy and interpretability of the model. It has been shown that variable selection using LASSO is oftentimes more accurate compared to traditional methods such as stepwise procedures [27]. Furthermore, given the existing ambiguity about how to merge LASSO models across multiple imputed datasets, and given the relatively small amount of missing data in our dataset, we opted for a single imputation exclusively for the LASSO models. In addition, for simplicity, potential clustering within our data was not considered in these analyses. For each response variable, a LASSO model was developed according to the following procedure. First, the optimal shrinkage parameter of each model was determined by performing a grid search. For each lambda in the grid, bootstrapping with 50 iterations was performed and the average predictive performance (i.e., mean squared error) across all out-of-bag samples was calculated using the machine learning in R “mlr” package [28]. Second, the lambda value that produced the highest out-of-bag predictive performance was selected as the optimal lambda value and used for the final LASSO model fitted to the whole sample.

# Results

When the results were tested in a smaller subset including only one-dose per study (doses close to 100 µg LSD, N = 213, see Supplementary Figure S2) as a sensitivity analysis, only the VAS “any drug effect” (β = 0.22, p_c_ = 0.019), “good drug effect” (β = 0.20, p_c_ = 0.034), 5D-ASC “Changed Meaning of Percepts” (β = 0.25, p_c_ = 0.008), MAP (β = 0.23, p_c_ = 0.012), and LSD blood concentration (β = 0.41, p_c_ < 0.001) remained significantly predicted by the now small differences in the drug doses (see Supplementary Figure S1). Lower CYP2D6 activity still predicted higher LSD plasma concentration (β = -0.28, p_c_ = 0.001) and now even more strongly predicted the 5D-ASC total score (β = -0.26, p_c_ = 0.008). Previous experiences of hallucinogens still predicted overall alteration of consciousness (5D-ASC, β = -0.23, p_c_ = 0.011) and the subscales “Anxious Ego Dissolution”, “Vigilance Reduction”, “Disembodiment”, and “Impaired Control and Cognition”, as well as MEQ30’s “Transcendence of Space and Time” (β = -0.23, p_c_ = 0.013, β = -0.25, p_c_ = 0.008, β = -0.22, p_c_ = 0.020, β = -0.24, p_c_ = 0.009, and β = -0.23, p_c_ = 0.024, respectively). Age was still predictive for “bad drug effects” (β = 0.25, p_c_ = 0.008). Most of the predictive effects of the predrug mood state were still significant. In particular, “General Well-Being” still predicted “good drug effects” (β = 0.30, p_c_ = 0.001), 5D-ASC total score (β = 0.32, p_c_ < 0.001), and “Oceanic Boundlessness” (β = 0.31, p_c_ < 0.001). "Emotional Excitability" and “Anxiety” remained associated with heart rate (β = 0.25, p_corrected_ = 0.009 and β = 0.31, p_c_ = 0.001, respectively) and body temperature ("Emotional Excitability", β = 0.21, p_corrected_ = 0.036), while "Performance-related activity" and "Extraversion" became less important overall. “Anxiety” still predicted 5D-ASC “Anxiety” (β = 0.24, p_corrected_ = 0.009). The character trait “Openness to Experience” still predicted “Oceanic Boundlessness” (β = 0.20, p_corrected_ = 0.034) and “Insightfulness” (β = 0.26, p_corrected_ = 0.008) on the 5D-ASC and the total score (β = 0.23, p_corrected_ = 0.019) and “Mystical” (β = 0.24, p_corrected_ = 0.017) on the MEQ30. The trait “Extraversion” additionally predicted “Elementary Imagery” (β = 0.19, p_corrected_ = 0.049). Sex only still predicted body temperature (β = -0.21, p_corrected_ = 0.035). Body weight and setting (MRI) were no longer predictive for any response variables.

**Supplementary Table S1**

Detailed overview of the studies included in the pooled analysis.

| **Supplementary Table S1**. Study population | | |  |  |  |  |  |  |  |  |  |  |  |  |  |  |  |  |  |  |  |  |  |
| --- | --- | --- | --- | --- | --- | --- | --- | --- | --- | --- | --- | --- | --- | --- | --- | --- | --- | --- | --- | --- | --- | --- | --- |
|  |  |  |  |  |  |  |  |  |  |  |  |  |  |  |  |  |  |  |  |  |  |  |  |
|  | Clinical Trial # | | NCT01878942 |  | NCT02308969 |  | NCT03019822 |  | NCT03321136 | | | |  | NCT03604744 | |  | NCT04227756 |  | NCT04516902 |  | NCT04558294 |  | NCT04865653 |
|  | Study # | | 1 |  | 2 |  | 3 |  | 4 | | | |  | 5 | |  | 6 |  | 7 |  | 8 |  | 9 |
|  | Declared Dose, μg | | 200 |  | 100 |  | 100 |  | 25 | 50 | 100 | 200 |  | 100 | 200 |  | 100 |  | 100 |  | 100 |  | 100 |
|  | calculated dose^a^/quality control, μg (LSD base) | | 158 |  | 56 |  | 96 |  | 25.7 | 51.4 | 98.7 | 197.4 |  | 84.5 | 169 |  | 92.5 |  | 92.5 |  | 92.5 |  | 83.1 |
|  | Subjects, N in this study | | 16 |  | 24 |  | 27 |  | 16 | 20 | 16 | 19 |  | 30 | 29 |  | 32 |  | 24 |  | 24 |  | 20 |
|  | MRI measurement | | no |  | yes |  | yes |  | no | no | no | no |  | no | no |  | yes |  | no |  | no |  | no |
|  | Female [%] | | 8 [50] |  | 12 [50] |  | 14 [52] |  | 8 [50] | 11 [55] | 8 [50] | 10 [53] |  | 16 [53] | 15 [52] |  | 16 [50] |  | 12 [50] |  | 12 [50] |  | 10 [50] |
|  | CYP 2D6 non-functional, N | | 1 |  | 1 |  | 2 |  | 3 | 3 | 3 | 3 |  | 0 | 0 |  | 3 |  | 1 |  | 1 |  | NA |
|  | Bodyweight, kg | | 73 ± 13 |  | 68 ± 8 |  | 71 ± 12 |  | 69 ± 15 | 69 ± 15 | 69 ± 15 | 68 ± 15 |  | 72 ± 12 | 72 ± 12 |  | 71 ± 10 |  | 68 ± 10 |  | 71 ± 14 |  | 70 ± 12 |
|  |  | Range, kg | 52 - 98 |  | 55 - 85 |  | 55 - 97 |  | 50 - 93 | 50 - 93 | 50 - 93 | 50 - 93 |  | 55 - 104 | 55 - 104 |  | 52 - 90 |  | 51 - 85 |  | 50 - 102 |  | 50 - 93 |
|  | Age, years | | 29 ± 6 |  | 33 ± 11 |  | 28 ± 4 |  | 29 ± 6 | 29 ± 6 | 29 ± 6 | 29 ± 6 |  | 36 ± 10 | 36 ± 10 |  | 29 ± 4 |  | 30 ± 7 |  | 34 ± 12 |  | 37.2 ± 11 |
|  |  | Range, years | 25 - 52 |  | 25 - 60 |  | 25 - 45 |  | 25 - 52 | 25 - 52 | 25 - 52 | 25 - 52 |  | 25 - 56 | 25 - 54 |  | 25 - 44 |  | 25 - 54 |  | 25 - 64 |  | 25 - 57 |
|  | Subjects with previous hallucinogens experience, N [%] | | 7 [44] |  | 4 [17] |  | 5 [19] |  | 6 [38] | 8 [40] | 6 [38] | 7 [37] |  | 14 [47] | 14 [48] |  | 20 [63] |  | 12 [50] |  | 11 [46] |  | 11 [55] |
|  |  | Range, N times taken hallucinogens^b^ | 0 - 3 |  | 0 - 2 |  | 0 - 2 |  | 0 - 3 | 0 - 3 | 0 - 3 | 0 - 3 |  | 0 - 5 | 0 - 5 |  | 0 - 10 |  | 0 - 6 |  | 0 - 6 |  | 0 - 10 |
|  | Last measurements after substance administartion, h | | 24 |  | 24 |  | 11.5 |  | 24 | | | |  | 24 | |  | 24 |  | 24 |  | 12 |  | 24 |
|  |  |  |  |  |  |  |  |  |  |  |  |  |  |  |  |  |  |  |  |  |  |  |  |
| MRI, Magnetic resonance imaging; CYP, cytochrome P450 | | | | | | | | | | | | | | | | | | | | | | | |
| ^a^The estimated real dose for Study #1 and #2 was linearly calculated by taking quality controlled data from study #3 and #4 and compare actually measured pharmacokinetic data between the different studies (LSD AUC∞). ^b^Lifetime hallucinogen consumption prior to participation in the respective study. | | | | | | | | | | | | | | | | | | | | | | | |

**Supplementary Table S2**

Describtive statistics of all predictor variables.

| **Supplementary Table S2**. Descriptive statistics of all predictor variables | | |
| --- | --- | --- |
|  |  |  |
| Predictor variable | Mean ± SD | Range |
| LSD Dose, µg | 100 ± 44 | 25.7 - 197.4 |
| Female [%] | 51 |  |
| Age, years | 31.6 ± 8.9 | 25 - 64 |
| Body weight, kg | 70.2 ± 12 | 50 - 104 |
| CYP2D6 Genetic Activity Score^a^ | 1.5 ± 0.6 | 0 - 4 |
| MRI [%] | 39 |  |
| Lifetime use of hallucinogens^b^ | 1.8 ± 2.0 | 0 - 11 |
| Performance-Related Activity (AMRS) | 15.9 ± 4.4 | 8 - 28 |
| General Inactivation (AMRS) | 15.3 ± 3.5 | 12 - 28 |
| Extraversion (AMRS) | 8.7 ± 2.4 | 4 - 16 |
| Introversion (AMRS) | 4.5 ± 0.9 | 4 - 8 |
| General Well-Being (AMRS) | 18.1 ± 4.5 | 8 - 30 |
| Emotional Excitability (AMRS) | 14.7 ± 2.3 | 12 - 24 |
| Anxiety (AMRS) | 8.5 ± 1.3 | 8 - 16 |
| Neuroticism (NEO-FFI) | 1.3 ± 0.5 | 0.167 - 3 |
| Extraversion (NEO-FFI) | 2.5 ± 0.5 | 1.167 - 3.5 |
| Openness to Experience (NEO-FFI) | 2.9 ± 0.5 | 1 - 3.917 |
| Agreeableness (NEO-FFI) | 2.8 ± 0.4 | 1.5 - 3.83 |
| Conscientiousness (NEO-FFI) | 2.7 ± 0.5 | 1 - 4 |
|  |  |  |
| N = 213; MRI, Magnetic resonance imaging; CYP, cytochrome P450; VAS, visual analog scale; AMRS, Adjective Mood Rating Scale; NEO-FFI, NEO Five-Factor Inventory; ^a^N = 191; ^b^N = 297. | | |

**Supplementary Table S3**

Tabular view of independent and dependent variables with percent missing data.

| **Supplementary Table S3**. Missing Data and Proportion Imputed using MICE | |
| --- | --- |
|  |  |
| variable | Proportion imputed (%) |
| Drug dose | 0 |
| Sex | 0 |
| Age | 0 |
| Weight | 0 |
| CYP2D6 Genetic Activity Score | ^a^0 |
| MRI | 0 |
| Number of times taken LSD | 0 |
| Performance-related activity (AMRS) | 1.3 |
| General inactivation (AMRS) | 1.7 |
| Extroversion (AMRS) | 1.3 |
| Introversion (AMRS) | 1.3 |
| General well being (AMRS) | 1.7 |
| Emotional excitability (AMRS) | 1.7 |
| Anxiety (AMRS) | 1.3 |
| Neuroticism (NEO-FFI) | 4.0 |
| Extroversion (NEO-FFI) | 4.0 |
| Openness to experience (NEO-FFI) | 4.0 |
| Agreeableness (NEO-FFI) | 4.0 |
| Conscientiousness (NEO-FFI) | 4.0 |
| Any drug effects (VAS) | ^b^0 |
| Bad drug effects (VAS) | ^b^0 |
| Good drug effects (VAS) | ^b^0 |
| Heart rate | ^c^3.3 |
| Mean arterial blood pressure | ^c^3.3 |
| Body temperature | ^c^3.3 |
| Altered State of Conciousness (5D-ASC) | ^b^0 |
| Oceanic Boundlessness (5D-ASC) | ^b^0 |
| Anxious Ego Dissolution (5D-ASC) | ^b^0 |
| Visionary Restructuralization (5D-ASC) | ^b^0 |
| Auditory Alteration (5D-ASC) | ^b^0 |
| Vigilance Reduction (5D-ASC) | ^b^0 |
| Experience of Unity (5D-ASC) | ^b^0 |
| Spiritual Experience (5D-ASC) | ^b^0 |
| Blissful State (5D-ASC) | ^b^0 |
| Insightfulness (5D-ASC) | ^b^0 |
| Disembodiment (5D-ASC) | ^b^0 |
| Impaired Control and Cognition (5D-ASC) | ^b^0 |
| Anxiety (5D-ASC) | ^b^0 |
| Complex Imagery (5D-ASC) | ^b^0 |
| Elementary Imagery (5D-ASC) | ^b^0 |
| Audio Visual Synesthesiae (5D-ASC) | ^b^0 |
| Changed Meaning of percepts (5D-ASC) | ^b^0 |
| Total Mystical Experience Score (MEQ30) | ^d^0 |
| Mystical (MEQ30) | ^d^0 |
| Positive Mood (MEQ30) | ^d^0 |
| Transcendence of Space & Time (MEQ30) | ^d^0 |
| Ineffability (MEQ30) | ^d^0 |
| LSD Blood Plasma Concentration (AUC) | 3.4 |
|  |  |
| MICE, Multivariate Imputation via Chained Equations package in R (22); MRI, Magnetic resonance imaging; CYP, cytochrome P450; VAS, visual analog scale; AMRS, Adjective Mood Rating Scale; NEO-FFI, NEO Five-Factor Inventory; 5D-ASC, five dimensional Altered States of Conciousness; MEQ30, 30-item Mystical Effects Questionnaire; AUC, Area under the curve | |
| ^a^10% were missing but not imputed | |
| ^b^11.1% were missing from the placebo session, they were replaced with 0 to simulate the difference from placebo. | |
| ^c^11.1% of the placebo parameters were missing, but only 3.3% of the difference from placebo was imputed; Study #8 was not included because of the missing placebo condition. | |
| ^d^8.1% were missing from Study #2 but were not imputed; Study #2 was not included in the analysis of MEQ30. | |

**Supplementary Table S4**

| **Supplementary Table S4.** Statistical estimates underlying Figure 1 | | | | | | | | |  |
| --- | --- | --- | --- | --- | --- | --- | --- | --- | --- |
| **Predictor variable** | **Response variable** | **β-Estimate** | | **p-value** | | **corrected p-value** | | **semi-partial R^2^** | |
| Age | Bad drug effects (VAS) | 0.232 | <0.001*** | | 0.001** | | 0.08 | |  |
| Age | Heart Rate | -0.228 | <0.001*** | | 0.008** | | 0.06 | |  |
| Anxiety (AMRS) | Anxiety (5D-ASC) | 0.244 | <0.001*** | | <0.001*** | | 0.07 | |  |
| Anxiety (AMRS) | Bad drug effects (VAS) | 0.166 | 0.004** | | 0.021* | | 0.07 | |  |
| Anxiety (AMRS) | Anxious Ego Dissolution (5D-ASC) | 0.165 | 0.003** | | 0.020* | | 0.09 | |  |
| Anxiety (AMRS) | Heart Rate | 0.227 | <0.001*** | | <0.001*** | | 0.05 | |  |
| Anxiety (AMRS) | Mean Arterial Blood Pressure | 0.169 | 0.004** | | 0.021* | | 0.03 | |  |
| Body weight | Bad drug effects (VAS) | -0.163 | 0.008** | | 0.038* | | 0.08 | |  |
| CYP2D6 Genetic Activity Score | Altered State of Conciousness (5D-ASC) | -0.205 | 0.003** | | 0.018* | | 0.11 | |  |
| CYP2D6 Genetic Activity Score | Anxiety (5D-ASC) | -0.235 | <0.001*** | | 0.002** | | 0.07 | |  |
| CYP2D6 Genetic Activity Score | Auditory Alteration (5D-ASC) | -0.212 | 0.005** | | 0.025* | | 0.04 | |  |
| CYP2D6 Genetic Activity Score | Anxious Ego Dissolution (5D-ASC) | -0.239 | <0.001*** | | 0.002** | | 0.09 | |  |
| CYP2D6 Genetic Activity Score | Impaired Control and Cognition (5D-ASC) | -0.206 | 0.003** | | 0.019* | | 0.06 | |  |
| CYP2D6 Genetic Activity Score | LSD Blood Plasma Concentration (AUC) | -0.191 | <0.001*** | | 0.002** | | 0.40 | |  |
| CYP2D6 Genetic Activity Score | Spiritual Experience (5D-ASC) | -0.208 | 0.005** | | 0.026* | | 0.04 | |  |
| Drug dose | Altered State of Conciousness (5D-ASC) | 0.319 | <0.001*** | | <0.001*** | | 0.10 | |  |
| Drug dose | Anxiety (5D-ASC) | 0.280 | <0.001*** | | <0.001*** | | 0.08 | |  |
| Drug dose | Any drug effects (VAS) | 0.410 | <0.001*** | | <0.001*** | | 0.17 | |  |
| Drug dose | Audio Visual Synesthesiae (5D-ASC) | 0.282 | <0.001*** | | <0.001*** | | 0.08 | |  |
| Drug dose | Auditory Alteration (5D-ASC) | 0.171 | <0.001*** | | <0.001*** | | 0.03 | |  |
| Drug dose | Bad drug effects (VAS) | 0.269 | <0.001*** | | <0.001*** | | 0.07 | |  |
| Drug dose | Blissful State (5D-ASC) | 0.143 | 0.001** | | 0.009** | | 0.02 | |  |
| Drug dose | Changed Meaning of Percepts (5D-ASC) | 0.222 | <0.001*** | | <0.001*** | | 0.05 | |  |
| Drug dose | Complex Imagery (5D-ASC) | 0.288 | <0.001*** | | <0.001*** | | 0.08 | |  |
| Drug dose | Disembodiment (5D-ASC) | 0.202 | <0.001*** | | <0.001*** | | 0.04 | |  |
| Drug dose | Anxious Ego Dissolution (5D-ASC) | 0.313 | <0.001*** | | <0.001*** | | 0.10 | |  |
| Drug dose | Elementary Imagery (5D-ASC) | 0.291 | <0.001*** | | <0.001*** | | 0.08 | |  |
| Drug dose | Experience of Unity (5D-ASC) | 0.249 | <0.001*** | | <0.001*** | | 0.06 | |  |
| Drug dose | Good drug effects (VAS) | 0.283 | <0.001*** | | <0.001*** | | 0.08 | |  |
| Drug dose | Heart Rate | 0.230 | <0.001*** | | <0.001*** | | 0.05 | |  |
| Drug dose | Impaired Control and Cognition (5D-ASC) | 0.261 | <0.001*** | | <0.001*** | | 0.07 | |  |
| Drug dose | Ineffability (MEQ30) | 0.404 | <0.001*** | | <0.001*** | | 0.16 | |  |
| Drug dose | Insightfulness (5D-ASC) | 0.171 | <0.001*** | | <0.001*** | | 0.03 | |  |
| Drug dose | LSD Blood Plasma Concentration (AUC) | 0.626 | <0.001*** | | <0.001*** | | 0.40 | |  |
| Drug dose | Mean Arterial Blood Pressure | 0.148 | <0.001*** | | 0.006** | | 0.02 | |  |
| Drug dose | Mystical (MEQ30) | 0.180 | <0.001*** | | <0.001*** | | 0.03 | |  |
| Drug dose | Oceanic Boundlessness (5D-ASC) | 0.244 | <0.001*** | | <0.001*** | | 0.06 | |  |
| Drug dose | Positive Mood (MEQ30) | 0.181 | <0.001*** | | 0.002** | | 0.03 | |  |
| Drug dose | Spiritual Experience (5D-ASC) | 0.145 | <0.001*** | | 0.001** | | 0.02 | |  |
| Drug dose | Total Mystical Experience Score (MEQ30) | 0.273 | <0.001*** | | <0.001*** | | 0.08 | |  |
| Drug dose | Transcendence of Space & Time (MEQ30) | 0.363 | <0.001*** | | <0.001*** | | 0.13 | |  |
| Drug dose | Vigilance Reduction (5D-ASC) | 0.174 | <0.001*** | | <0.001*** | | 0.03 | |  |
| Drug dose | Visionary Restructuralization (5D-ASC) | 0.334 | <0.001*** | | <0.001*** | | 0.11 | |  |
| Emotional Excitability (AMRS) | Altered State of Conciousness (5D-ASC) | 0.184 | <0.001*** | | 0.006** | | 0.11 | |  |
| Emotional Excitability (AMRS) | Anxiety (5D-ASC) | 0.186 | 0.001** | | 0.009** | | 0.09 | |  |
| Emotional Excitability (AMRS) | Blissful State (5D-ASC) | 0.204 | <0.001*** | | 0.006** | | 0.04 | |  |
| Emotional Excitability (AMRS) | Body Temperature | 0.196 | <0.001*** | | 0.004** | | 0.05 | |  |
| Emotional Excitability (AMRS) | Anxious Ego Dissolution (5D-ASC) | 0.225 | <0.001*** | | 0.001** | | 0.11 | |  |
| Emotional Excitability (AMRS) | Experience of Unity (5D-ASC) | 0.216 | <0.001*** | | 0.002** | | 0.07 | |  |
| Emotional Excitability (AMRS) | Good drug effects (VAS) | 0.154 | 0.006** | | 0.029* | | 0.09 | |  |
| Emotional Excitability (AMRS) | Heart Rate | 0.201 | 0.001** | | 0.009** | | 0.06 | |  |
| Emotional Excitability (AMRS) | Impaired Control and Cognition (5D-ASC) | 0.203 | <0.001*** | | 0.005** | | 0.08 | |  |
| Emotional Excitability (AMRS) | Oceanic Boundlessness (5D-ASC) | 0.198 | <0.001*** | | 0.004** | | 0.07 | |  |
| Emotional Excitability (AMRS) | Positive Mood (MEQ30) | 0.159 | 0.009** | | 0.040* | | 0.04 | |  |
| Emotional Excitability (AMRS) | Spiritual Experience (5D-ASC) | 0.147 | 0.011* | | 0.045* | | 0.02 | |  |
| Emotional Excitability (AMRS) | Total Mystical Experience Score (MEQ30) | 0.162 | 0.007** | | 0.033* | | 0.08 | |  |
| Emotional Excitability (AMRS) | Transcendence of Space & Time (MEQ30) | 0.162 | 0.006** | | 0.029* | | 0.14 | |  |
| Emotional Excitability (AMRS) | Vigilance Reduction (5D-ASC) | 0.158 | 0.006** | | 0.028* | | 0.03 | |  |
| Extraversion (AMRS) | Altered State of Conciousness (5D-ASC) | 0.237 | <0.001*** | | <0.001*** | | 0.11 | |  |
| Extraversion (AMRS) | Audio Visual Synesthesiae (5D-ASC) | 0.225 | <0.001*** | | 0.002** | | 0.08 | |  |
| Extraversion (AMRS) | Auditory Alteration (5D-ASC) | 0.188 | 0.002** | | 0.015* | | 0.03 | |  |
| Extraversion (AMRS) | Blissful State (5D-ASC) | 0.182 | 0.003** | | 0.019* | | 0.04 | |  |
| Extraversion (AMRS) | Disembodiment (5D-ASC) | 0.156 | 0.011* | | 0.045* | | 0.04 | |  |
| Extraversion (AMRS) | Anxious Ego Dissolution (5D-ASC) | 0.166 | 0.005** | | 0.025* | | 0.10 | |  |
| Extraversion (AMRS) | Elementary Imagery (5D-ASC) | 0.162 | 0.007** | | 0.034* | | 0.08 | |  |
| Extraversion (AMRS) | Experience of Unity (5D-ASC) | 0.201 | <0.001*** | | 0.006** | | 0.06 | |  |
| Extraversion (AMRS) | Good drug effects (VAS) | 0.202 | <0.001*** | | 0.005** | | 0.09 | |  |
| Extraversion (AMRS) | Insightfulness (5D-ASC) | 0.169 | 0.005** | | 0.027* | | 0.03 | |  |
| Extraversion (AMRS) | Mystical (MEQ30) | 0.176 | 0.006** | | 0.028* | | 0.03 | |  |
| Extraversion (AMRS) | Oceanic Boundlessness (5D-ASC) | 0.217 | <0.001*** | | 0.002** | | 0.06 | |  |
| Extraversion (AMRS) | Spiritual Experience (5D-ASC) | 0.169 | 0.005** | | 0.027* | | 0.02 | |  |
| Extraversion (AMRS) | Total Mystical Experience Score (MEQ30) | 0.173 | 0.005** | | 0.027* | | 0.08 | |  |
| Extraversion (AMRS) | Transcendence of Space & Time (MEQ30) | 0.186 | 0.002** | | 0.014* | | 0.14 | |  |
| Extraversion (AMRS) | Vigilance Reduction (5D-ASC) | 0.177 | 0.003** | | 0.018* | | 0.03 | |  |
| Extraversion (AMRS) | Visionary Restructuralization (5D-ASC) | 0.213 | <0.001*** | | 0.002** | | 0.12 | |  |
| Extraversion (NEO-FFI) | Altered State of Conciousness (5D-ASC) | 0.174 | 0.005** | | 0.027* | | 0.10 | |  |
| Extraversion (NEO-FFI) | Audio Visual Synesthesiae (5D-ASC) | 0.197 | 0.002** | | 0.012* | | 0.08 | |  |
| Extraversion (NEO-FFI) | Auditory Alteration (5D-ASC) | 0.240 | <0.001*** | | 0.004** | | 0.05 | |  |
| Extraversion (NEO-FFI) | Changed Meaning of Percepts (5D-ASC) | 0.158 | 0.013* | | 0.050* | | 0.05 | |  |
| Extraversion (NEO-FFI) | Visionary Restructuralization (5D-ASC) | 0.188 | 0.002** | | 0.015* | | 0.11 | |  |
| Female | Body Temperature | -0.194 | 0.002** | | 0.016* | | 0.05 | |  |
| Female | Experience of Unity (5D-ASC) | -0.172 | 0.009** | | 0.038* | | 0.06 | |  |
| Female | Good drug effects (VAS) | -0.165 | 0.008** | | 0.036* | | 0.08 | |  |
| Female | Insightfulness (5D-ASC) | -0.174 | 0.009** | | 0.038* | | 0.03 | |  |
| Female | Positive Mood (MEQ30) | -0.172 | 0.009** | | 0.038* | | 0.03 | |  |
| Female | Spiritual Experience (5D-ASC) | -0.172 | 0.012* | | 0.048* | | 0.03 | |  |
| General Inactivation (AMRS) | Heart Rate | 0.166 | 0.007** | | 0.031* | | 0.05 | |  |
| General Well-Being (AMRS) | Altered State of Conciousness (5D-ASC) | 0.285 | <0.001*** | | <0.001*** | | 0.12 | |  |
| General Well-Being (AMRS) | Anxiety (5D-ASC) | 0.171 | 0.003** | | 0.020* | | 0.08 | |  |
| General Well-Being (AMRS) | Any drug effects (VAS) | 0.160 | 0.004** | | 0.021* | | 0.19 | |  |
| General Well-Being (AMRS) | Audio Visual Synesthesiae (5D-ASC) | 0.176 | 0.003** | | 0.020* | | 0.09 | |  |
| General Well-Being (AMRS) | Auditory Alteration (5D-ASC) | 0.164 | 0.009** | | 0.038* | | 0.03 | |  |
| General Well-Being (AMRS) | Blissful State (5D-ASC) | 0.209 | <0.001*** | | 0.006** | | 0.05 | |  |
| General Well-Being (AMRS) | Changed Meaning of Percepts (5D-ASC) | 0.153 | 0.012* | | 0.048* | | 0.05 | |  |
| General Well-Being (AMRS) | Complex Imagery (5D-ASC) | 0.177 | 0.003** | | 0.020* | | 0.09 | |  |
| General Well-Being (AMRS) | Disembodiment (5D-ASC) | 0.197 | 0.001** | | 0.010* | | 0.05 | |  |
| General Well-Being (AMRS) | Anxious Ego Dissolution (5D-ASC) | 0.245 | <0.001*** | | <0.001*** | | 0.11 | |  |
| General Well-Being (AMRS) | Elementary Imagery (5D-ASC) | 0.200 | <0.001*** | | 0.008** | | 0.09 | |  |
| General Well-Being (AMRS) | Experience of Unity (5D-ASC) | 0.224 | <0.001*** | | 0.002** | | 0.07 | |  |
| General Well-Being (AMRS) | Good drug effects (VAS) | 0.270 | <0.001*** | | <0.001*** | | 0.10 | |  |
| General Well-Being (AMRS) | Impaired Control and Cognition (5D-ASC) | 0.239 | <0.001*** | | 0.001** | | 0.08 | |  |
| General Well-Being (AMRS) | Ineffability (MEQ30) | 0.168 | 0.004** | | 0.021* | | 0.18 | |  |
| General Well-Being (AMRS) | Insightfulness (5D-ASC) | 0.199 | 0.001** | | 0.009** | | 0.04 | |  |
| General Well-Being (AMRS) | Mystical (MEQ30) | 0.186 | 0.004** | | 0.022* | | 0.04 | |  |
| General Well-Being (AMRS) | Oceanic Boundlessness (5D-ASC) | 0.252 | <0.001*** | | <0.001*** | | 0.07 | |  |
| General Well-Being (AMRS) | Spiritual Experience (5D-ASC) | 0.214 | <0.001*** | | 0.005** | | 0.04 | |  |
| General Well-Being (AMRS) | Total Mystical Experience Score (MEQ30) | 0.201 | 0.001** | | 0.009** | | 0.08 | |  |
| General Well-Being (AMRS) | Transcendence of Space & Time (MEQ30) | 0.206 | <0.001*** | | 0.006** | | 0.14 | |  |
| General Well-Being (AMRS) | Vigilance Reduction (5D-ASC) | 0.248 | <0.001*** | | <0.001*** | | 0.07 | |  |
| General Well-Being (AMRS) | Visionary Restructuralization (5D-ASC) | 0.235 | <0.001*** | | <0.001*** | | 0.13 | |  |
| Lifetime use of hallucinogens | Altered State of Conciousness (5D-ASC) | -0.196 | <0.001*** | | 0.004** | | 0.11 | |  |
| Lifetime use of hallucinogens | Changed Meaning of Percepts (5D-ASC) | -0.196 | <0.001*** | | 0.006** | | 0.06 | |  |
| Lifetime use of hallucinogens | Disembodiment (5D-ASC) | -0.182 | 0.002** | | 0.013* | | 0.05 | |  |
| Lifetime use of hallucinogens | Anxious Ego Dissolution (5D-ASC) | -0.193 | <0.001*** | | 0.006** | | 0.11 | |  |
| Lifetime use of hallucinogens | Impaired Control and Cognition (5D-ASC) | -0.209 | <0.001*** | | 0.003** | | 0.08 | |  |
| Lifetime use of hallucinogens | Transcendence of Space & Time (MEQ30) | -0.162 | 0.005** | | 0.026* | | 0.14 | |  |
| Lifetime use of hallucinogens | Vigilance Reduction (5D-ASC) | -0.212 | <0.001*** | | 0.002** | | 0.04 | |  |
| Lifetime use of hallucinogens | Visionary Restructuralization (5D-ASC) | -0.157 | 0.005** | | 0.025* | | 0.12 | |  |
| MRI | Impaired Control and Cognition (5D-ASC) | 0.173 | 0.005** | | 0.025* | | 0.07 | |  |
| Neuroticism (NEO-FFI) | Ineffability (MEQ30) | 0.157 | 0.008** | | 0.036* | | 0.17 | |  |
| Openness to Experience (NEO-FFI) | Blissful State (5D-ASC) | 0.157 | 0.012* | | 0.050* | | 0.02 | |  |
| Openness to Experience (NEO-FFI) | Experience of Unity (5D-ASC) | 0.170 | 0.008** | | 0.038* | | 0.06 | |  |
| Openness to Experience (NEO-FFI) | Insightfulness (5D-ASC) | 0.216 | <0.001*** | | 0.006** | | 0.05 | |  |
| Openness to Experience (NEO-FFI) | Mystical (MEQ30) | 0.220 | <0.001*** | | 0.008** | | 0.04 | |  |
| Openness to Experience (NEO-FFI) | Oceanic Boundlessness (5D-ASC) | 0.186 | 0.003** | | 0.017* | | 0.06 | |  |
| Openness to Experience (NEO-FFI) | Positive Mood (MEQ30) | 0.213 | <0.001*** | | 0.008** | | 0.04 | |  |
| Openness to Experience (NEO-FFI) | Spiritual Experience (5D-ASC) | 0.166 | 0.011* | | 0.045* | | 0.02 | |  |
| Openness to Experience (NEO-FFI) | Total Mystical Experience Score (MEQ30) | 0.199 | 0.002** | | 0.014* | | 0.08 | |  |
| Performance-Related Activity (AMRS) | Altered State of Conciousness (5D-ASC) | 0.176 | 0.002** | | 0.015* | | 0.11 | |  |
| Performance-Related Activity (AMRS) | Anxiety (5D-ASC) | 0.159 | 0.006** | | 0.030* | | 0.08 | |  |
| Performance-Related Activity (AMRS) | Any drug effects (VAS) | 0.154 | 0.005** | | 0.027* | | 0.18 | |  |
| Performance-Related Activity (AMRS) | Anxious Ego Dissolution (5D-ASC) | 0.163 | 0.006** | | 0.028* | | 0.10 | |  |
| Performance-Related Activity (AMRS) | Experience of Unity (5D-ASC) | 0.154 | 0.010* | | 0.043* | | 0.06 | |  |
| Performance-Related Activity (AMRS) | Good drug effects (VAS) | 0.205 | <0.001*** | | 0.004** | | 0.09 | |  |
| Performance-Related Activity (AMRS) | Insightfulness (5D-ASC) | 0.158 | 0.010** | | 0.042* | | 0.03 | |  |
| Performance-Related Activity (AMRS) | Oceanic Boundlessness (5D-ASC) | 0.168 | 0.004** | | 0.024* | | 0.06 | |  |
| Performance-Related Activity (AMRS) | Spiritual Experience (5D-ASC) | 0.180 | 0.003** | | 0.020* | | 0.03 | |  |
| Performance-Related Activity (AMRS) | Vigilance Reduction (5D-ASC) | 0.187 | 0.002** | | 0.013* | | 0.03 | |  |
| MRI, Magnetic resonance imaging; CYP, cytochrome P450; VAS, visual analog scale; AMRS, Adjective Mood Rating Scale; NEO-FFI, NEO Five-Factor Inventory; 5D-ASC, five dimensional Altered States of Conciousness; MEQ30, 30-item Mystical Effects Questionnaire; AUC, Area under the curve. | | | | | | | | |  |

**
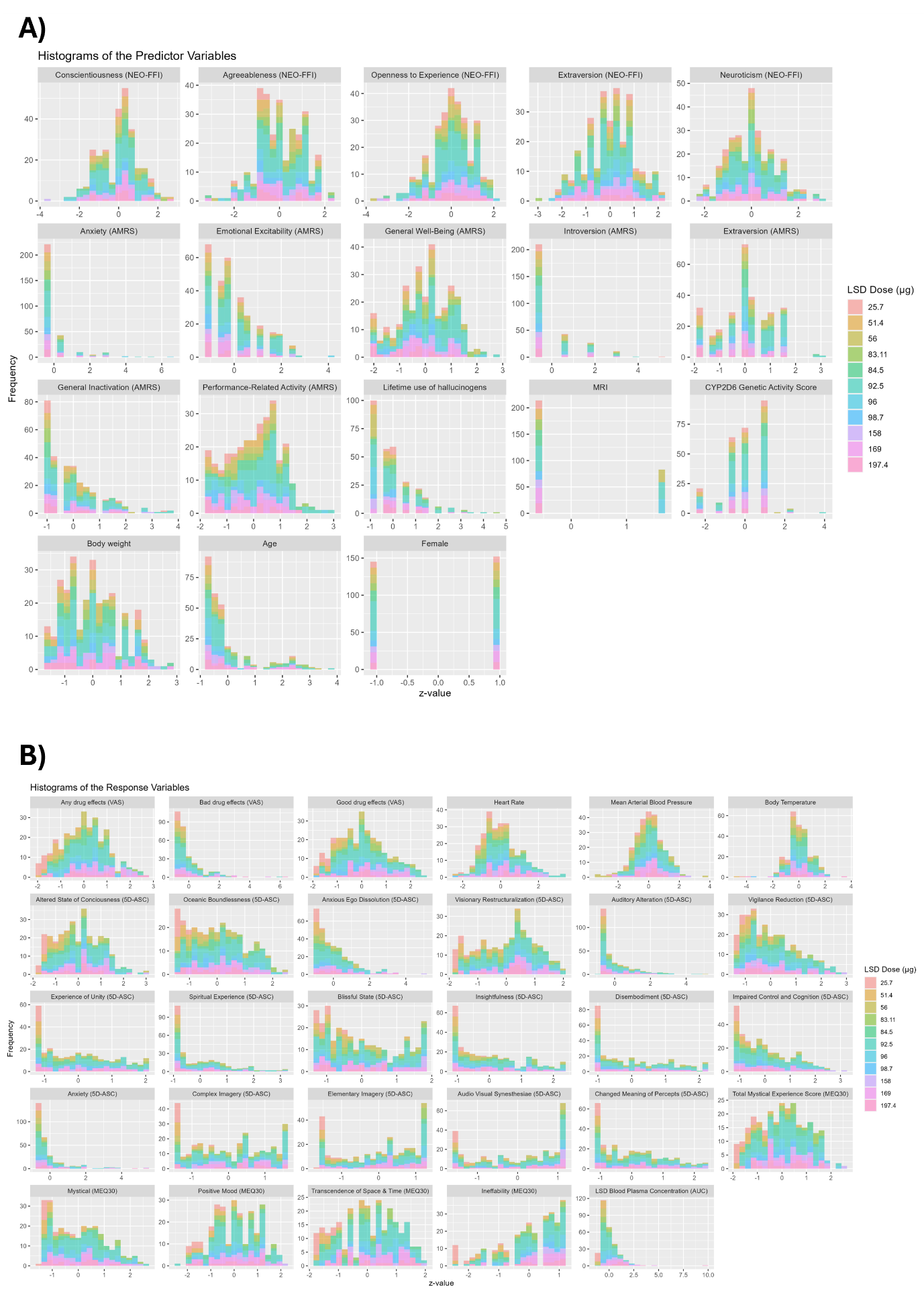
**

**Supplementary Figure S1 (A-B)**

Histograms of the predictor **(A)** and response **(B)** variables in the dataset. Coloration displays the different analytically confirmed LSD doses. The peak effect was used for the physiological effects. CYP, cytochrome P450. MRI, Magnetic resonance imaging. VAS, visual analog scale (Area under the effect-time curve 0-11.5 h). AMRS, Adjective Mood Rating Scale. NEO-FFI, NEO Five-Factor Inventory. 5D-ASC, five dimensional Altered States of Consciousness. MEQ30, 30-item Mystical Effects Questionnaire. AUC, Area under the curve from 0 - ∞ h.


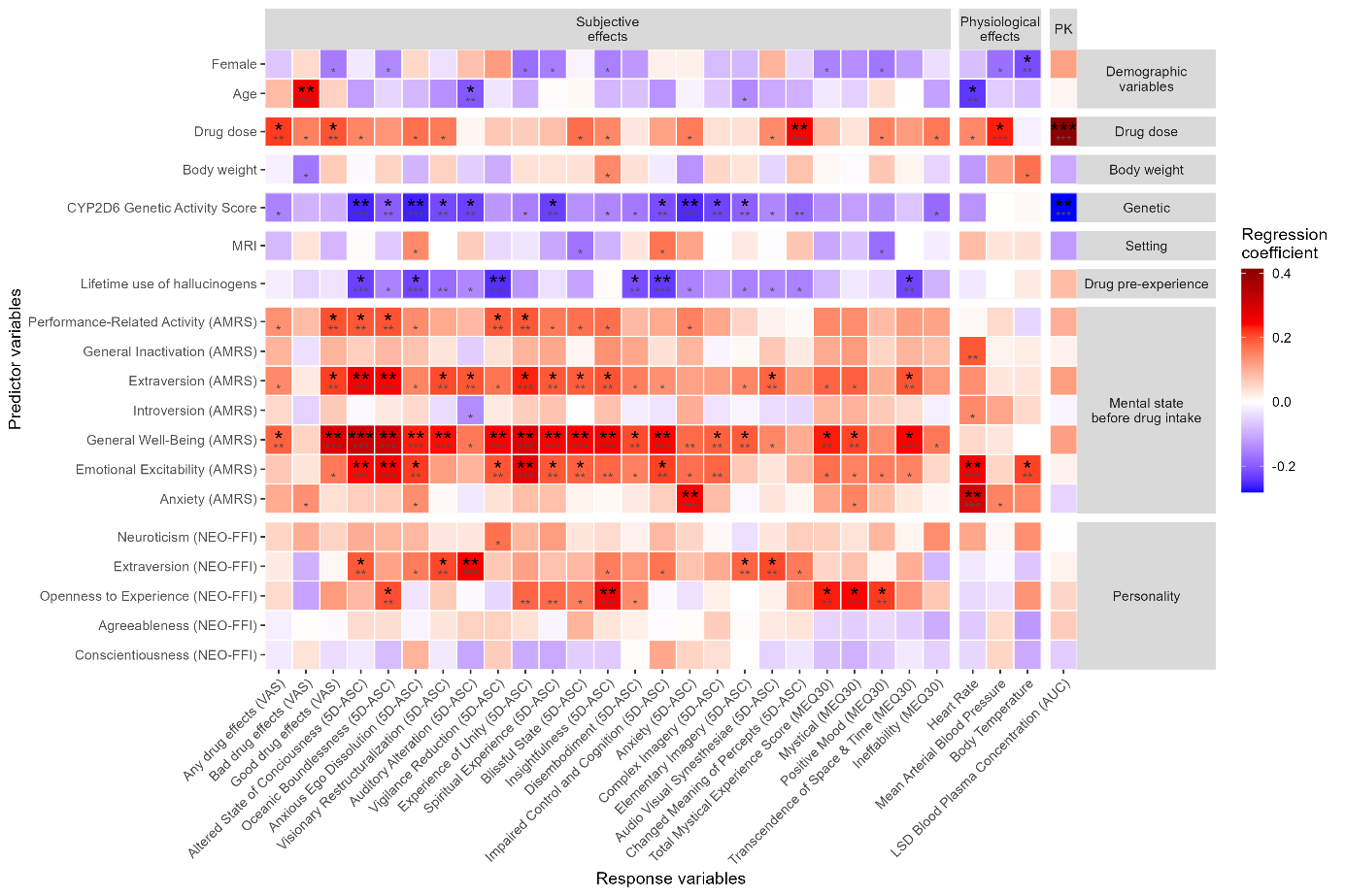


**Supplementary Figure S2**

Standardized regression coefficients and statistical significance of each predictor variable in the linear mixed effects models adjusting for drug dose (except drug dose) in a supportive sensitivity analysis using only data from one LSD session per study (N = 213). The data used are the difference between the LSD session with the drug dose closest to 100 µg LSD and the respective placebo session. Smaller asterisks show the uncorrected statistical significance. Bigger asterisks show the significance after correction for multiple testing across all 19 * 29 = 551 significance tests using the Benjamini-Hochberg procedure [29]. **p*<0.05, ***p*<0.01, ****p*<0.001. The peak effect was used for the physiological effects. CYP, cytochrome P450. MRI, Magnetic resonance imaging. VAS, visual analog scale (Area under the effect-time curve 0-11.5 h). AMRS, Adjective Mood Rating Scale. NEO-FFI, NEO Five-Factor Inventory. 5D-ASC, five dimensional Altered States of Consciousness. MEQ30, 30-item Mystical Effects Questionnaire. AUC, Area under the curve from 0 - ∞ h.


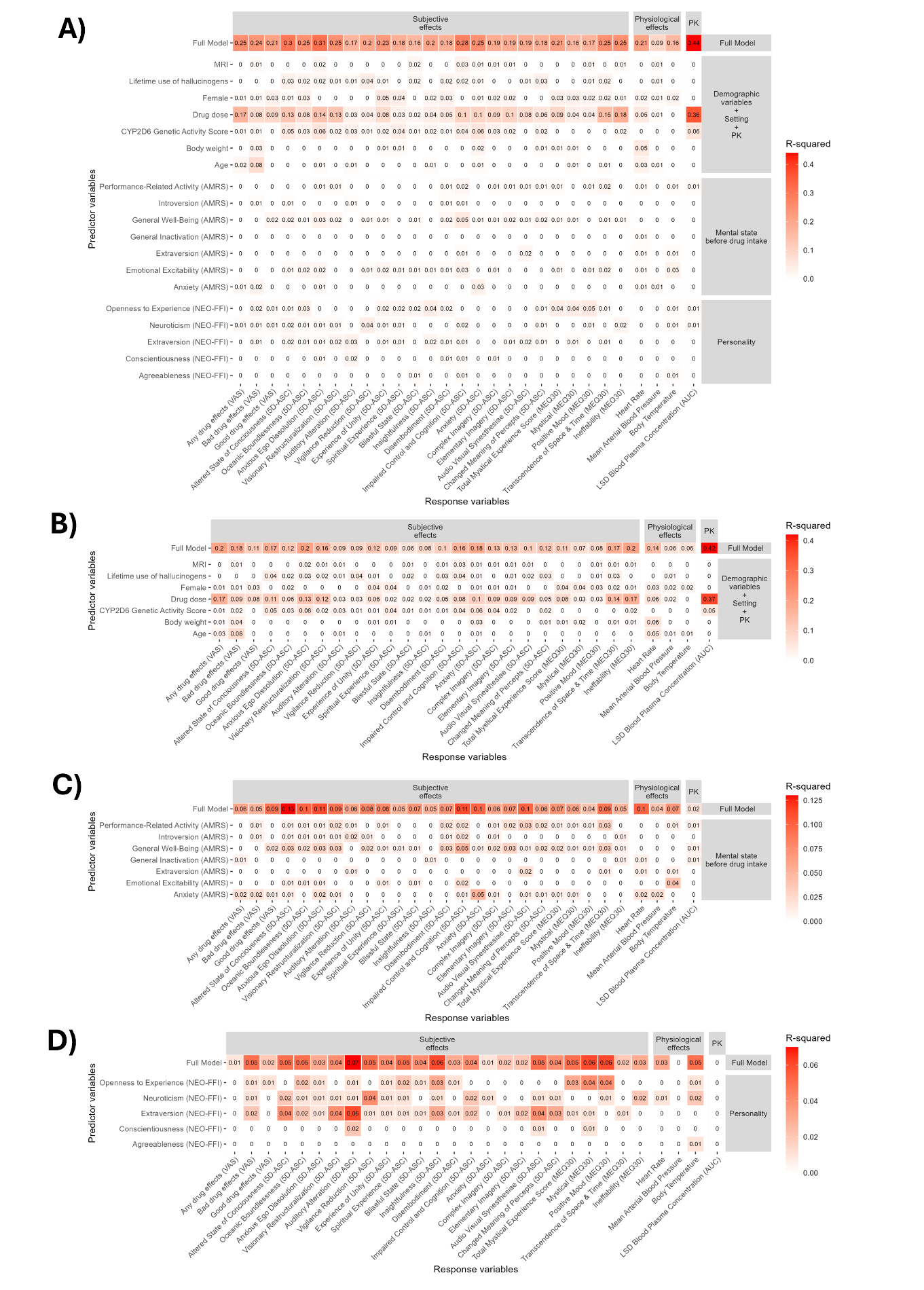


**Supplementary Figure S3 (A-D)**

R-squared values of the predictors in an overall **(A)** and subsets **(B-D)** models. Model with Demographic, Setting and PK variables only **(B)**; Model with mental state before drug intake only **(C)**, Model with personality traits only **(D)**. “0” means a value < 0.005. To ensure that there was no severe multicollinearity between predictor variables, the variance inflation factor was calculated and remained < 5 for all predictors. The peak effect was used for the physiological effects. CYP, cytochrome P450. MRI, Magnetic resonance imaging. VAS, visual analog scale (Area under the effect-time curve 0-11.5 h). AMRS, Adjective Mood Rating Scale. NEO-FFI, NEO Five-Factor Inventory. 5D-ASC, five dimensional Altered States of Consciousness. MEQ30, 30-item Mystical Effects Questionnaire. AUC, Area under the curve from 0 - ∞ h.


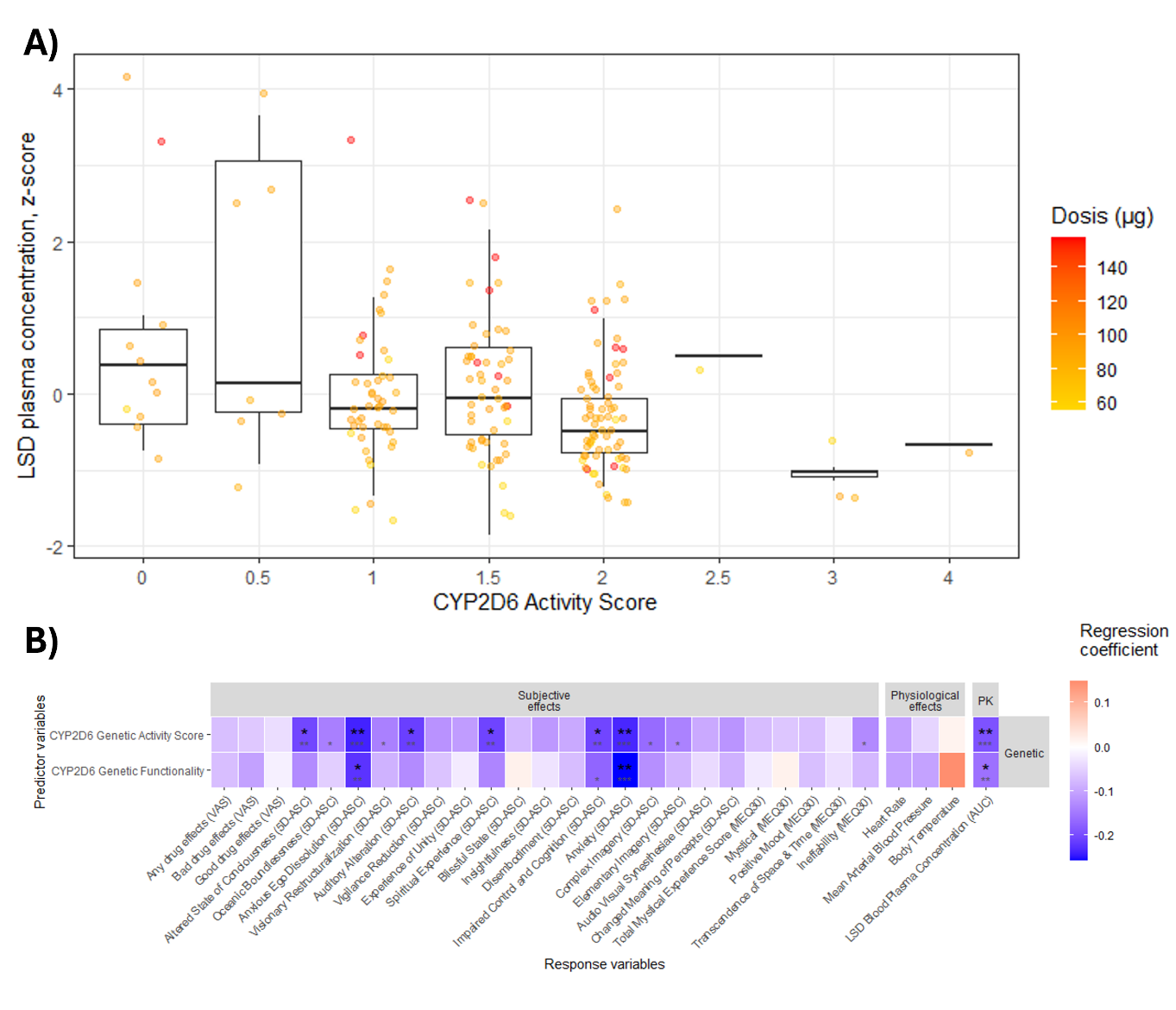


**Supplementary Figure S4 (A-B)**

Detailed description of the CYP2D6 Activity Scores according to Gaedigk et al. [8]. Boxplot of the CYP2D6 Activity Score and LSD plasma concentration **(A).** Model results comparison between binary CYP2D6 functionality (activity score 0 = non-functional genotype, >0 = functional [7]) and CYP2D6 Activity Score (**B**). N = 267. The peak effect was used for the physiological effects. CYP, cytochrome P450. MRI, Magnetic resonance imaging. VAS, visual analog scale (Area under the effect-time curve 0-11.5 h). AMRS, Adjective Mood Rating Scale. NEO-FFI, NEO Five-Factor Inventory. 5D-ASC, five dimensional Altered States of Consciousness. MEQ30, 30-item Mystical Effects Questionnaire. AUC, Area under the curve from 0 - ∞ h.

**References**

1. Liechti ME, Gamma A, and Vollenweider FX, *Gender differences in the subjective effects of MDMA,* Psychopharmacology (Berl), 2001. **154**(2): p. 161-8.

2. Vizeli P and Liechti ME, *Safety pharmacology of acute MDMA administration in healthy subjects,* J Psychopharmacol, 2017. **31**(5): p. 576-588.

3. Studerus E, Gamma A, Kometer M, and Vollenweider FX, *Prediction of psilocybin response in healthy volunteers,* PLoS One, 2012. **7**(2): p. e30800.

4. Wagmann L, Richter LHJ, Kehl T, Wack F, Bergstrand MP, Brandt SD, et al., *In vitro metabolic fate of nine LSD-based new psychoactive substances and their analytical detectability in different urinary screening procedures,* Anal Bioanal Chem, 2019. **411**(19): p. 4751-4763.

5. Luethi D, Hoener MC, Krahenbuhl S, Liechti ME, and Duthaler U, *Cytochrome P450 enzymes contribute to the metabolism of LSD to nor-LSD and 2-oxo-3-hydroxy-LSD: Implications for clinical LSD use,* Biochem Pharmacol, 2019. **164**: p. 129-138.

6. Gaedigk A, *Complexities of CYP2D6 gene analysis and interpretation,* Int Rev Psychiatry, 2013. **25**(5): p. 534-53.

7. Vizeli P, Straumann I, Holze F, Schmid Y, Dolder PC, and Liechti ME, *Genetic influence of CYP2D6 on pharmacokinetics and acute subjective effects of LSD in a pooled analysis,* Sci Rep, 2021. **11**(1): p. 10851.

8. Gaedigk A, Simon SD, Pearce RE, Bradford LD, Kennedy MJ, and Leeder JS, *The CYP2D6 activity score: translating genotype information into a qualitative measure of phenotype,* Clin Pharmacol Ther, 2008. **83**(2): p. 234-42.

9. Caudle KE, Sangkuhl K, Whirl-Carrillo M, Swen JJ, Haidar CE, Klein TE, et al., *Standardizing CYP2D6 Genotype to Phenotype Translation: Consensus Recommendations from the Clinical Pharmacogenetics Implementation Consortium and Dutch Pharmacogenetics Working Group,* Clin Transl Sci, 2020. **13**(1): p. 116-124.

10. Studerus E, Vizeli P, Harder S, Ley L, and Liechti ME, *Prediction of MDMA response in healthy humans: a pooled analysis of placebo-controlled studies,* J Psychopharmacol, 2021. **35**(5): p. 556-565.

11. Janke W and Debus G, *Die Eigenschaftswörterliste.* 1978, Göttingen.: Hogrefe.

12. Borkenau P and Ostendorf F, eds. *NEO-Fünf-Faktoren-Inventar (NEO-FFI) nach Costa und McCrae*. 2nd ed. 2008, Hogrefe: Göttingen.

13. Dittrich A, *The standardized psychometric assessment of altered states of consciousness (ASCs) in humans,* Pharmacopsychiatry, 1998. **31 (Suppl 2)**: p. 80-4.

14. Studerus E, Gamma A, and Vollenweider FX, *Psychometric evaluation of the altered states of consciousness rating scale (OAV),* PLoS One, 2010. **5**(8): p. e12412.

15. Barrett FS, Johnson MW, and Griffiths RR, *Validation of the revised Mystical Experience Questionnaire in experimental sessions with psilocybin,* J Psychopharmacol, 2015. **29**(11): p. 1182-90.

16. Liechti ME, Dolder PC, and Schmid Y, *Alterations in conciousness and mystical-type experiences after acute LSD in humans,* Psychopharmacology, 2017. **234**: p. 1499-1510.

17. Griffiths RR, Richards WA, McCann U, and Jesse R, *Psilocybin can occasion mystical-type experiences having substantial and sustained personal meaning and spiritual significance,* Psychopharmacology (Berl), 2006. **187**(3): p. 268-83; discussion 284-292.

18. Griffiths RR, Johnson MW, Richards WA, Richards BD, McCann U, and Jesse R, *Psilocybin occasioned mystical-type experiences: immediate and persisting dose-related effects,* Psychopharmacology, 2011. **218**(4): p. 649-65.

19. Griffiths R, Richards W, Johnson M, McCann U, and Jesse R, *Mystical-type experiences occasioned by psilocybin mediate the attribution of personal meaning and spiritual significance 14 months later,* J Psychopharmacol, 2008. **22**(6): p. 621-32.

20. Ross S, Bossis A, Guss J, Agin-Liebes G, Malone T, Cohen B, et al., *Rapid and sustained symptom reduction following psilocybin treatment for anxiety and depression in patients with life-threatening cancer: a randomized controlled trial,* J Psychopharmacol, 2016. **30**(12): p. 1165-1180.

21. Garcia-Romeu A, Griffiths RR, and Johnson MW, *Psilocybin-occasioned mystical experiences in the treatment of tobacco addiction,* Curr Drug Abuse Rev, 2014. **7**(3): p. 157-64.

22. Holze F, Duthaler U, Vizeli P, Muller F, Borgwardt S, and Liechti ME, *Pharmacokinetics and subjective effects of a novel oral LSD formulation in healthy subjects,* Br J Clin Pharmacol, 2019. **85**: p. 1474-83.

23. Buuren Sv and Groothuis-Oudshoorn K, *mice: Multivariate imputation by chained equations in R,* Journal of statistical software, 2010: p. 1-68.

24. Enders CK, *Applied missing data analysis*. Methodology in the social sciences. 2010, New York: Guilford Press. xv, 377 p.

25. Graham JW, Olchowski AE, and Gilreath TD, *How many imputations are really needed? Some practical clarifications of multiple imputation theory,* Prev Sci, 2007. **8**(3): p. 206-13.

26. Little RJ and Rubin DB, *Statistical analysis with missing data*. Vol. 793. 2019: Wiley.

27. Tibshirani R, *The lasso method for variable selection in the Cox model,* Statistics in medicine, 1997. **16**(4): p. 385-395.

28. Bischl B, Lang M, Kotthoff L, Schiffner J, Richter J, Studerus E, et al., *mlr: Machine Learning in R,* The Journal of Machine Learning Research, 2016. **17**(1): p. 5938-5942.

29. Benjamini Y and Hochberg Y, *Controlling the false discovery rate: a practical and powerful approach to multiple testing,* Journal of the Royal statistical society: series B (Methodological), 1995. **57**(1): p. 289-300.
